# Sustainable grain production growth of farmland–A role of agricultural socialized services

**DOI:** 10.1016/j.heliyon.2024.e26755

**Published:** 2024-02-21

**Authors:** Aimin Wu, Ehsan Elahi, Fengtong Cao, Mohammad Yusuf, Mohammad Ilyas Abro

**Affiliations:** a School of Economics, Shandong University of Technology (SDUT), Zibo, 255049, Shandong, People's Republic of China; b Clean Energy Technologies Research Institute (CETRI), Process Systems Engineering, Faculty of Engineering and Applied Science, University of Regina, Regina, SK, 3737 Wascana Parkway, S4S 0A2, Canada; c Centre of Research Impact and Outcome, Chitkara University Institute of Engineering and Technology, Chitkara University, Punjab, India; d Department of Basic Science and Humanities, Dawood University of Engineering and Technology, Karachi City, Pakistan

**Keywords:** Land, Food, Agriculture, Farmers, China

## Abstract

The main aim of this study is to examine the evolving landscape of agricultural socialized services and their impact on the consistent growth of grain production in China. Using panel data from 2007 to 2020, we employ the Entropy Method to gauge the dynamic changes in agricultural socialized services that have contributed to the steady increase in grain production. The research methods include static panel, mediator, and threshold regression models to investigate the effects and mechanisms underpinning the improvement of agricultural socialized services on grain production growth. The empirical findings demonstrate a significantly positive correlation between enhanced agricultural socialized services, such as means of production services, sci-tech information services, and social public services, and increased grain production. This positive impact persists even with limited grain production resources. A mediating effect was identified, whereby agricultural socialized services indirectly stimulate grain production growth by encouraging large-scale agricultural land management. Furthermore, threshold analysis indicates the presence of a single threshold effect linked to the level of agricultural socialization services. This threshold effect plays a pivotal role in the relationship between large-scale agricultural management and steady grain production growth. The study suggests an enhancement of agricultural socialized services can promote sustained growth in grain production.

## 1 Introduction

Following longstanding wisdom asserting the paramount importance of food security for all, it is pivotal to recognize the centrality of grain production. This assertion holds particular significance not only for a nation's economic landscape but also for the holistic well-being of its population [[1], [2], [3], [4], [5], [6]]. Indeed, it serves as the bedrock upon which national economic development and social stability are built [7]. In recent years, amidst China's comprehensive reform efforts, the relentless march of new industrialization and urbanization, the surging food demands within China, and the escalating risks to global grain production, China's food security faces a multitude of challenges. Since farm production is affected by managerial, institutional, and climatic factors [[8], [9], [10], [11]], ensuring stable grain production has become imperative for global and national food security. This commitment is underscored by the December 2022 release of the Outline of the Strategic Plan for Expanding Domestic Demand (2022–2035) by the Central Committee and the State Council, which explicitly emphasizes China's need to ensure food security and promote stable grain production.

The pursuit of stable grain production not only underscores the importance of maintaining a consistent upward trajectory in grain production but also places a strong emphasis on the welfare of farmers [12]. Securing farmers' income stands as the foundational defense in consolidating national food security [[13], [14], [15], [16]]. Currently, China's grain production landscape still bears the hallmark of a great country with weak agriculture. China's grain production depends heavily on small-scale farmers, with around 230 million rural households involved in lease-holding arrangements for agricultural land. This vast number of small farming operations highlights the vital role of family-based agriculture in achieving ample nationwide grain outputs. Sustaining robust grain harvests will require policies that support the productivity and profitability of the hundreds of millions of small farms across rural China [17].

For an extended period, these small farmers have primarily resorted to substantial inputs of fertilizers, pesticides, and other production materials to enhance grain production. While this approach has contributed to production growth, it has also exhibited pronounced unsustainability issues, consequences include the degradation of arable land, diminishing grain quality, declining grain income, and dwindling motivation among farmers [18]. Additionally, the role of agricultural means of production, influenced by factors such as the loss of rural labor force and tightening agricultural resources, has seen its positive effects erode, potentially leading to imbalances between input and output and thereby affecting production stability [19].

In recent years, with the continuous innovation of agricultural production methods in China, grain production has promoted the increase of grain output through the large-scale agricultural land management formed by the circulation of agricultural land. Large-scale agricultural land management refers to a management method in which qualified farmers collectively manage a certain amount of land and promote the efficiency of grain production by fully utilizing the capabilities of production factors [20]. Although this model has significantly improved the output rate of land, there are still a series of problems such as high land rental costs and low labor productivity in grain production [21]. Consequently, it cannot adequately guarantee the consistent growth of grain production. Considering these challenges, China seeks a renewed impetus for grain production to ensure steady growth and attain food security.

Agricultural socialized services refer to various services provided by various social and economic organizations to meet the needs of agricultural production before, during, and after production, including agricultural production material services, agricultural financial services, agricultural science, and technology information services [22]. Agricultural socialized services have emerged as an important means of promoting agricultural industrialization and modernization. By enabling specialization, outsourcing, and updated techniques, these services enhance productivity across numerous roles [23]. Moreover, agricultural socialized services bolster the systematic nature of agricultural production, bridging the gap between small-scale agricultural production and the vast agricultural marketplace [24]. Acknowledging the transformative potential inherent in agricultural socialized services, the Ministry of Agriculture and Rural Affairs issued the Guidance on Accelerating the Development of Agricultural Socialized Services in 2021. This directive underscores the pivotal role of such services in igniting farmers' enthusiasm for production and fostering heightened agricultural productivity [25].

Nevertheless, China's overall level of agricultural socialized services remains in its infancy, marked by a low proportion of agricultural businesses and conspicuous regional disparities [26]. An optimal strategy to secure steady grain production may well involve assisting small farmers, who dominate grain production, in optimizing the allocation of resources and facilitating large-scale agricultural land management, thereby fostering a ‘grain-oriented’ approach to agricultural land [27]. Consequently, it is imperative to investigate whether high-level agricultural socialized services can indeed augment grain production, particularly delving into the primary mechanisms driving this impact.

The study is intricately intertwined with two distinct bodies of literature. The first strand primarily investigates the impact of agricultural socialized services on the augmentation of grain production. The introduction of agricultural socialized services emerges as a significant driving force propelling the integration of China's small-holder grain production system into the trajectory of modern agriculture [28]. Operating as an innovative productivity tool, agricultural socialized services not only enhance the operational efficiency of the grain industry [29] but also elevate the technical efficiency of grain production [30] and optimize the allocation of grain production resources and conditions [31]. Consequently, agricultural socialized services harbor the potential to boost grain total factor productivity and foster the high-quality development of grain production [32].

The second facet of literature explores the influence of agricultural socialized services on farmers' income. It contends that these services constitute an effective avenue for augmenting farmers' earnings [33]. Through the strategic utilization of market resources, these services efficiently align production factors with grain farmers, resulting in reduced production costs and a simultaneous increase in income from grain management [34]. This dual role effectively balances the equation, elevating farmers' income while concurrently decreasing expenditures [35].

The current study focuses on examining the isolated effects of agricultural socialized services on either grain production or farmers' income, often neglecting their combined impact. Building upon the groundwork laid by these two strands of literature, this study aims to investigate two interconnected aspects. First, it explores the influence of agricultural socialized services on the sustained growth of grain yield, considering the amalgamation of grain yield and income. Second, the study delves into the transmission mechanisms and regional disparities in the impact of agricultural socialized services on grain yield. To achieve this, empirical tests are conducted using data from 31 provincial-level regions in China spanning from 2007 to 2020. The overarching goal is to illuminate the role of enhanced agricultural socialized services in fostering regional and steady growth in grain production. There are five main contributions of the study.

1. *Comprehensive Examination of Agricultural Socialized Services:* This study undertakes a comprehensive investigation into the evolving landscape of agricultural socialized services and their pivotal role in the context of grain production in China. It contributes to the understanding of the interplay between these services and the grain sector, an area of paramount importance in the realm of food policy.
2. *Empirical Rigor through Extensive Temporal Analysis:* Through the meticulous utilization of panel data spanning a notable duration from 2007 to 2020, this study achieves a high degree of empirical rigor. This extended temporal coverage allows for a thorough and nuanced assessment of the relationship between agricultural socialized services and grain production, thereby enhancing the scholarly robustness of the findings.
3. *Quantified Positive Correlation Between Services and Grain Production:* This study quantifies a significantly positive correlation between the augmentation of agricultural socialized services and the amplification of grain production. This quantification establishes a vital empirical foundation, delivering actionable insights to policymakers and stakeholders vested in the augmentation of grain production for food security.
4. *Mechanistic Insights into Positive Impact:* In addition to establishing a correlation, this study delves into the intricate mechanisms underpinning the favorable impact of agricultural socialized services on grain production. By identifying specific dimensions such as agricultural means of production services, agricultural sci-tech information services, and social public services, it offers nuanced insights into the multifaceted nature of this relationship.
5. *Addressing Regional Disparities and Threshold Effects*: This study encompasses an exploration of regional disparities in the influence of agricultural socialized services on grain production. It also unveils threshold effects associated with the level of agricultural socialization services. These analytical dimensions contribute to a refined understanding of how the impact of these services may exhibit variability across different geographical contexts and levels of development.

These contributions collectively render this study a noteworthy addition to the academic discourse surrounding food policy, agricultural dynamics, and grain production. The findings hold significant implications for informed policy formulation and strategic initiatives aimed at bolstering food security, not only in the context of China but also for regions confronting analogous challenges. To achieve the aims of the study, the whole article is organized as follows: The next section provides the theoretical framework and hypotheses (section 2). Section 3 belongs to the research methods to achieve the objectives of the study. Section 4 explains the findings. Moreover, section 5 presents the summary of the findings with policy implications.

## 2 Theoretical framework and hypotheses

### 2.1 Direct impact: enhancing agricultural socialized services for steady growth in grain production

Enhancing agricultural socialized services has a direct and positive influence on fostering the steady growth of grain production [36,37]. This impact arises from several key factors. Firstly, as agricultural production and management become more specialized and intricately divided, the improvement of agricultural socialized services effectively bridges grain production with market-oriented operations. This facilitates the engagement of service providers from diverse sectors across the entire food supply chain, encouraging a shift in grain production from being primarily internalized to a more externalized approach [38]. This shift leads to a granular specialization of labor within grain production, thereby promoting a specialized division of labor. Consequently, this specialization streamlines grain production, optimizes production modes, and enhances the overall productivity of grain production [39]. Consequently, this approach helps mitigate the issues caused by land fragmentation and labor shortages. Additionally, farmers can obtain production materials through agricultural socialized services, facilitating large-scale mechanized operations. This enhances production efficiency in two ways: 1) it directly improves agricultural operations via coordination and scale, and 2) it substantially reduces input costs through collective material procurement. Together, these effects significantly increase the overall efficiency of grain production and management. Additionally, the specialization effect, catalyzed by improved agricultural socialized services, further promotes the standardization of the entire grain industry chain [40]. This transition towards increased standardization naturally generates economies of scale, resulting in not only positive reinforcement of improved agricultural socialized services' efficiency but also reduced service costs for farmers. This alleviates the pressure faced by farmers in grain production and management, simultaneously enhancing grain production efficiency and income, ultimately contributing to the advancement of grain production [41,42].

Secondly, as the entire grain business industry chain encompasses agricultural materials, storage, processing, trade, and finance, the improvement of agricultural socialized services can stimulate the integration and coordination of production factors such as capital, technology, and labor within the agricultural market [43]. This integration strengthens the synergy between various components of the grain industry chain, effectively leveraging complementary strengths and resources to achieve overall coordinated development of the grain industry chain [44]. This synergy enhances the optimization of resource allocation throughout the grain industry, effectively lowering the threshold for farmers to engage in the entire grain industry chain [45]. This facilitates the farmer's ability to overcome obstacles related to production factors and management, providing the necessary conditions for achieving steady growth in grain production. Therefore, given in Table 1, we proposed the hypothesis H1.

**Table 1:** Hypotheses of the study.

H1	Improvement in agricultural socialized services can promote steady growth in grain production.
H2	Improving agricultural socialized services can drive large-scale agricultural land management, indirectly contributing to steady growth in grain production.
H3	A threshold effect exists between large-scale land management and steady growth in grain production, contingent on the level of agricultural socialized services.

### 2.2 Indirect impacts: advancing agricultural socialized services and their role in fostering steady growth in grain production

The enhancement of agricultural socialized services can indirectly promote steady growth in grain production through several means, one of which is the promotion of large-scale farmland management. This promotion hinges on the substitution effect of production factors. Land, being the fundamental element of grain production, plays a pivotal role in achieving Pareto Optimality in resource allocation for grain production [46]. According to farmer behavior theory, farmers with ample capital and labor resources tend to leverage their surplus resources, beyond what is required for original grain production, to expand their grain production through land circulation [47]. Enhanced agricultural socialized services facilitate this process by providing farmers with high-quality agricultural machinery services, seed services, or land trusteeship services. Furthermore, service organizations can replace rural labor with “low-cost and high-efficiency” services such as mechanization, informatization, and standardization. These measures alleviate the constraints imposed by production factors on farmers seeking to engage in large-scale grain production, thus providing the practical conditions needed for large-scale agricultural land management [32].

The large-scale management of agricultural land, driven by agricultural socialized services, can improve the “grain-oriented” nature of farmland management and foster economies of scale in grain production, thereby stimulating scale economies effects [33]. This shift toward appropriate management leads to a reduction in the marginal cost of grain production, particularly concerning information, transaction, and decision-making costs. Consequently, this increase in efficiency boosts farmers' incomes. Furthermore, specialized production, encouraged by large-scale land management, enhances the quality of agricultural products, and mitigates the fragmented and decentralized production conditions typically associated with small-peasant economies. Grain production under a large-scale management model optimizes grain production patterns through scientific management. This scientific management improves the efficiency of information transfer to farmers, enabling better pre-production planning and enhancing the efficiency and effectiveness of grain production. Therefore, a research hypothesis (H2) has been proposed in Table 1.

### 2.3 Threshold effect of improving agricultural socialized service on steady growth in grain production

The steady growth in grain production, fostered by large-scale agricultural and management, heavily relies on robust support from agricultural socialized services [34,48]. Currently, China's strides in industrialization, urbanization, agricultural modernization, and marketization, along with structural transformations in the three major industries, have induced dynamic changes in various aspects of agricultural socialized services. These changes span levels, scales, structures, and resource conditions of agricultural socialized services across different regions. These disparities can lead to capital concentration during the initial stages of agricultural socialized services' expansion, which may result in disorderly growth, notably in the form of high service prices and diminished service efficiency. This phenomenon can significantly impact the effectiveness of large-scale land management, ultimately influencing the steady growth of grain production [49].

Specifically, in regions with advanced agricultural socialized services, steady growth in grain production tends to be easily achieved through large-scale land management [50]. The specialization of labor systems associated with higher levels of agricultural socialized services reduces constraints related to capital and technology for farmers and alleviates their production and operational burdens [51]. This encourages larger-scale grain production, triggering economies of scale and fostering steady growth. Conversely, regions with lower levels of agricultural socialized services may see reduced benefits in grain production due to a lack of specialized labor division. To maintain grain production, farmers engaged in large-scale management may need to invest more resources, thereby increasing production costs and reducing grain income. If the cost of grain remains unchanged, this could result in reduced grain yields. This underscores that in the dynamic landscape of agricultural socialized services, a homogeneous exploration of the impact of large-scale land management on grain yield may not suffice. Instead, a threshold effect might exist, dependent on the level of agricultural socialized services [52]. Therefore, it is imperative to investigate the non-linear characteristics that govern the relationship between large-scale land management and grain yield across different levels of agricultural socialized services. Therefore, a hypothesis (H3) can be proposed in Table 1.

## 3 Materials and methods

### 3.1 Model construction

#### 3.1.1 Reference model

Considering that there are large differences between agricultural socialized service and steady growth in grain production in time series and space, we construct a fixed-effect model to investigate whether the improvement of agricultural socialized service can promote steady growth in grain production.

(1) $$PI_{it}=\alpha_{0}+\alpha_{1}ASS_{it}+\theta X_{it}+\delta_{t}+u_{i}+\varepsilon_{it}$$

where PI means steady growth in grain production, ASS shows agricultural socialized service, and *X* is the control variable which includes population aging rate (age), natural disaster (dis), the level of opening up (op), the level of rural human capital (edu), and industrial structure adjustment (is). While *i*, and *t* represent the region and year, respectively. While $\delta_{t}$ is the time-fixed effect. The $u_{i}$ is the regional fixed effect, and the $\varepsilon_{it}$ is the random error term which is assumed to be normally distributed at zero mean value and constant variance [53].

*Mediating effect model*: To examine the mediating effect of the cultivated land scale between agricultural socialized services and grain yield, this study employs a stepwise regression test. The model construction, referencing the mediating effect test methodology outlined by Wen Zhong (2004), is detailed in equations (2), (3), (4) as follows:

(2) $$PI_{it}=\beta_{0}+\beta_{1}ASS_{it}+{\theta X}_{it}+\delta_{t}+u_{i}+\varepsilon_{it}$$

(3) $$SCA_{it}=\gamma_{0}+\gamma_{1}ASS_{it}+\theta X_{it}+\delta_{t}+u_{i}+\varepsilon_{it}$$

(4) $$PI_{it}=\mu_{0}+\mu_{1}ASS_{it}+\mu_{2}SCA_{it}+\theta X_{it}+\delta_{t}+u_{i}+\varepsilon_{it}$$

where the SCA is mediating variable that shows farmland scale management. The remaining variables are consistent with equation (1).

#### 3.1.2 Threshold effect model

To examine the multi-stage non-linear threshold effect of agricultural land scale management on grain yield across distinct levels of agricultural socialization services, this study utilizes the panel threshold model as proposed by Hansen [54]. The testing process involves the careful selection and application of the threshold model to facilitate thorough analysis.

(5) $$PI_{it}=\alpha_{0}+\alpha_{1}SCA_{it}\times I\left(ASS\leq \lambda_{1}\right)+\alpha_{2}SCA_{it}\times I\left(\lambda_{1}<ASS\leq \lambda_{2}\right)+\ldots +\alpha_{n}SCA_{it}\times I\left(\lambda_{n-1}<ASS\leq \lambda_{n}\right)+\alpha_{n+1}SCA_{it}\times I\left(ASS>\lambda_{n}\right)+{\theta X}_{it}+\varepsilon_{it}$$

In equation (5), ASS represents the threshold variable; λ shows the threshold estimate; I(∙) is the indicative function, taking 1 if true in parentheses and 0 otherwise; and the remaining variables are consistent with equation (1).

### 3.2 Descriptions of variables

#### 3.2.1 Explained variable

*Steady growth in grain production (PI):* The variables and indicators for study growth in grain production are given in Table 2. Since there is currently no direct statistical data available for this particular index, and considering that the core concept of steady growth in grain production involves the symbiosis between increasing grain production and improved income for farmers within the context of food security, this study draws upon the findings of Yao Chengsheng [55] regarding the quantity and stability of grain production growth, as well as research conducted by Qidi [56] on the enhancement of farmers' income. Adhering to principles of logical consistency and making the best use of available data, this study employs the entropy method to establish a comprehensive index system for measuring steady growth in grain production. This system encompasses two sub-indices: one tracking the steady growth in grain production and the other assessing the increase in income for farmers. Furthermore, to gauge the primary indicator – the stability of grain production– this study computes the volatility index for grain production, calculating its absolute value following the approach of Xiao Saili's methodology (2014).

**Table 2:** Indicators and variables for steady growth in grain production.

Target Layer	Criterion Layer	Index Layer	Formula	Attributes
Steady growth in grain production (PI)	Grain production growth	Stability of grain production	Absolute value of volatility index of grain yield	–
		Stability of grain production	Total grain production/grain acreage	+
	Income increases for the farmer	Engel coefficient in rural area	Per capita food consumption expenditure in rural areas or Per capita living expenditure in rural area	–
		Income of the farmer	The average net income of farmers engaged in agriculture in the year × Value of grain production	+

#### 3.2.2 Explanatory variables

*Agricultural socialized service (ASS):* The focal point of this study is ASS, with explicit reference to the research on the measurement of agricultural socialized services conducted by Liu et al. (2021) and Yang [57]. Adhering to principles of data availability and scientific rigor, this study adopts the methodology for constructing comprehensive indicators outlined by Han Miaomiao (2020). Agricultural socialized service, based on its service functions, is further categorized into four sub-item indicators: agricultural means of production service, agricultural science and technology service, agricultural financial circulation service, and agricultural social public service (Table 3). These indicators are evaluated using the entropy method, allowing for the measurement of annual agricultural socialized services in each region based on various criteria.

**Table 3:** Indicators and variables for agricultural socialized service.

Target Layer	Criterion Layer	Index Layer	Formula	Attributes
Agricultural Socialized Service（ASS)	Agricultural means of production services (am)	c1 Pesticide supply	Pesticide usage	+
		c2 Fertilizer supply	Pesticide plastic film usage	+
		c3 Agricultural plastic film supply	Agricultural technicians/Total rural population at year-end	+
	Agricultural science and technology information services (as)	c4 Generalization of agricultural science and technology information	Total power of agricultural machinery	+
		c5 Investment in agricultural technology	Total power of agricultural machinery	+
	Agricultural finance services (af)	c6 Insurance penetration	Agricultural insurance income/Total rural population at year-end	+
		c7 Agricultural loans penetration	Balance of loans related to agriculture/Total rural population at end of year	+
	Public services to the agricultural society (ps)	c8 Effective irrigation area	Irrigated area	+
		c9 Rural electricity supply	Rural electricity consumption	+
		c10 Rural highway coverage	(Total mileage of highways of the same grade-highways, primary and secondary highways + Total mileage except highway of the same grade)/Land area of each province	+

#### 3.2.3 Intervening variable

Agricultural land large-scale management (SCA) is defined based on the study conducted by Lu and Xiong [58]. It is represented by the ratio of crop sown area to agricultural practitioners, serving as an indicator of the extent of large-scale management of agricultural land. To ensure a comprehensive analysis while controlling for other significant factors that could influence the stable increase in grain yield, this study incorporates several control variables. These variables include:

*Regional population aging level (age):* This variable is defined as the proportion of the population aged 65 and above the total population in the region.

*Rural human capital (hc):* It represents the average level of education and schooling attained by the rural population in the region.

*The level of opening-up (op):* This variable measures the proportion of the total import and export volume of each province to its gross domestic product.

*The level of industrialization (il):* It quantifies the ratio of the added value of the secondary industry to the gross regional products.

These control variables are considered in the analysis to account for their potential effects on the steady increase in grain yield.

#### 3.2.4 Data specification

For the empirical analysis, we have employed panel data covering the period from 2007 to 2020, encompassing 31 provincial regions in China (excluding data from Hong Kong, Macao, and Taiwan). These data have been meticulously sourced from reputable references, including the China Statistical Yearbook, China Agricultural and Rural Statistical Yearbook, China Science and Technology Statistical Yearbook, along with other pertinent statistical sources. We've also made use of data from the official website of the National Bureau of Statistics and the Wind Database. In cases where data was missing, we've judiciously applied interpolation techniques to ensure data integrity. The study draws upon a range of essential variables extracted from these sources to underpin its empirical analysis (Table 4).

**Table 4:** Descriptive statistics of variables.

Variables	Symbols	Sample size	Average value	Standard deviation	Minimum value	Maximumvalue
Explained variable	PI	434	0.2186	0.1048	0.0273	0.5299
Explanatory variable	ASS	434	0.5219	0.1082	0.1083	0.8830
Intervening variable	SCA	434	0.0589	0.0442	0.0167	0.3299
Control variable	age	434	0.1003	0.0241	0.0482	0.1742
	hc	434	7.5346	0.8263	3.8038	9.6603
	op	434	0.4116	0.4311	0.0076	2.4629
	il	434	0.4205	0.0841	0.1583	0.6196

## 4 Results and discussion

### 4.1 Regression analysis of reference model

In this study, the F-test and Hausman test are used to screen the model. The results show that the F-test statistic is 227.54 (Prob > F = 0.0000), indicating that the individual effect is significant, and the mixed effect model should be rejected. Therefore, a two-way fixed effects model was used with regional and time effects. Fixed effects were chosen over random effects based on the Hausman specification test results. The test rejected the null hypothesis of no systematic difference in coefficients between fixed and random effects (Hausman stat = 18.98, p = 0.0019). This indicates that unobserved heterogeneity across provinces correlated with the explanatory variables biases random effects estimates. The fixed effects approach controls for this by allowing the intercept to differ per region, removing omitted variable bias from time-invariant characteristics. Thus, it enhances consistency in estimating the impact of agricultural socialized services on grain production growth. The formal test justification provides credibility for using fixed over random effects modeling.

As shown in Table 5, column (1) shows that the two-way fixed effect regression result without adding control variables, and the estimated coefficient of agricultural socialization service is significantly positive. Column (2) also shows that the estimated coefficient of agricultural socialization service is significantly positive, with an estimated coefficient of 0.3984. The regression results of the two models are consistent, indicating that the development of agricultural socialization services can help promote stable and increased production of grain, and hypothesis H1 is verified.

**Table 5:** Regression results of reference model.

Variables	FE						
	(1)	(2)	(3)	(4)	(5)	(6)	(7)
ASS	0. 3868*** (0. 1214)	0.3984*** (0.1086)					
am			1.5101*** (0.5274)				1.5020*** (0.5263)
as				0.1418** (0.3339)			0.0535* (0.0301)
af					−0.2176 (0.3732)		−0.1904 (0.3879)
ps						1.0787*** (0.3225)	0.9346*** (0.2872)
age		0.9273*** (0.3240)	0.8940*** (0.2822)	0.8577** (0.3339)	0.7791** 0.3491	0.7677** (0.3465)	0.8096*** (0.3161)
hc		−0.0057 (0.0189)	−0.0116 (0.0186)	−0.0047 (0.0199)	−0.0049 (0.0199)	−0.0011 (0.1049)	−0.0077 (0.1049)
op		−0.0246** (0.0101)	−0.0268*** (0.0085)	−0.0297*** (0.0093)	−0.0323*** (0.0108)	−0.0345*** (0.0092)	−0.0350*** (0.0077)
Il		0.1343 (0.0925)	0.1225 (0.0897)	0.1462 (0.1183)	0.1616 (0.1294)	0.1904* (0.1049)	0.1558* (0.0770)
Constant	0. 3580*** (0. 0229)	0.2638** (0.1271)	0.3040** (0.3040)	0.3215** (0.1435)	0.3356** (0.1545)	0.2432* (0.1368)	0.2280* (0.1435)
Regional fixed effect	Yes	Yes	Yes	Yes	Yes	Yes	Yes
Timel-Fixed effect	Yes	Yes	Yes	Yes	Yes	Yes	Yes
R^2^	0.4430	0.5371	0.3953	0.4187	0.3563	0.4825	0.4825
N	434	434	434	434	434	434	434

***, ** and * represent the level of significance of parameters at 1%, 5% and 10%, respectively. Standard errors are given in parentheses.

Considering that there are many fields involved in the explanatory variables, this part also takes the four sub-indicators of the comprehensive index of agricultural socialization service as explanatory variables for sub-regression analysis. Subsequent regression results of the sub-item indicators are outlined in columns (3) to (7) of Table 5, revealing that, apart from agricultural finance services, other services like agricultural capital services, agricultural scientific and technological information services, and social public services for agriculture also contribute effectively to the promotion of steady grain yield growth. This divergence may stem from issues in agricultural finance services, such as limited access to direct financing for agricultural goods and a lack of diversity in agricultural financial products, resulting in an overall lower development level.

The results also highlight the impact of control variables on grain yield. As indicated in column (2) of Table 5, the level of opening-up exerts an inhibitory effect on the steady growth of grain yield, whereas population aging promotes grain yield. However, industrialization and rural human capital appear to have no significant influence. These results can be attributed to several factors: Population aging accelerates agricultural technology improvement due to its anti-driving effect, resulting in increased grain yield. In highly open areas, there is fierce competition for productive factors between grain production and non-agricultural employment, leading to a decreased local grain income with the influx of imported grain into the local market. Additionally, low enthusiasm for grain production hampers grain yield growth. These conclusions align with the outcomes of other regression models in Table 5, reinforcing the credibility of the reference results and verifying hypothesis H1.

The positive impact of agricultural socialized services on grain production growth aligns with several key economic theories. Firstly, it resonates with Adam Smith's concepts on the productivity gains from specialization and division of labor. The improvement of these services allows for greater specialization along the grain production value chain, enabling productivity enhancements as predicted by Smith [59]. Secondly, the findings lend empirical support to Marschak & Andrews [60] seminal theory on the efficiency benefits of service-oriented firms in overcoming input rigidities faced by individual farmers. Agricultural socialized services similarly alleviate constraints and facilitate scale for higher agricultural output. Thirdly, the results validate Binswanger & Deininger [61] proposition regarding the role of specialized service agencies in aggregating demands, tailoring provisions to user needs, and promoting the adoption of improved farm practices– thereby catalyzing agricultural productivity growth. The significantly positive impact of agricultural socialized services is thus closely aligned with established economic principles around specialization, relieving input rigidities, aggregation solutions, and the systemic role of services in raising productivity. This theoretical grounding further elevates the policy relevance of the study findings.

### 4.2 Test for endogeneity and robustness

#### 4.2.1 Endogeneity test

Common endogenous problems include omitted variables, measurement errors, and endogeneity between variables. Although this study has attempted to mitigate the endogenous problems caused by omitted variables and measurement errors by selecting control variables and obtaining data from official databases such as the “China Statistical Yearbook”, the empirical model still faces the threat of endogeneity between variables. On the one hand, the improvement of agricultural socialization service levels can simplify the grain production process, optimize grain production methods, enhance total factor productivity of grain, help farmers overcome their own production bottlenecks and management disadvantages, and promote stable and increased grain production. On the other hand, the increase in stable and grain production levels means that farmers' income will increase, which will also promote the improvement of agricultural socialization service levels to a certain extent (Chen et al., 2020). The farmers who have more funds tend to invest in agricultural socialization services, such as purchasing more efficient agricultural machinery and adopting advanced grain production technologies. This will provide new opportunities and possibilities for improving agricultural socialization service levels. Therefore, there may be endogeneity problems of mutual causation between agricultural socialization services and stable and increased grain production. To mitigate potential issues related to reverse causality among variables and endogeneity problems stemming from unobserved factors, this study uses the System Generalized Method of Moments (SYS-GMM) for verification. Lagged values of the dependent variables were introduced as instrumental variables.

The SYS-GMM approach is used specifically to mitigate potential endogeneity issues between the variables. It utilizes instrumental variables and treats the explanatory variables as endogenous, allowing for control of unobserved heterogeneity and omitted variables. The lagged dependent variable is included as an instrument. The SYS-GMM method was chosen because of its strength in addressing endogeneity problems common in panel data analyses. Controlling for endogeneity enhances the robustness and reliability of estimating the true causal relationship between agricultural socialized services and grain production growth. However, the SYS-GMM approach also has some limitations. The use of internal instruments can be weak if the explanatory variables are too persistent. The proliferation of instruments may also overfit endogenous variables. To address these issues, we employ statistical tests for instrument validity and avoid instrument proliferation.

As indicated in column (1), the estimated coefficient for agricultural socialized services remained positive. The Arelleno Bond test results in column (1) showed that the p-value of residuals AR(1) is 0.016, dropped below the statistical significance level of 5%. Meanwhile, the p-value of AR(2) was found at 0.289, surpassing the statistical significance level of 10%. These findings suggest the presence of only first-order autocorrelation and the absence of second-order autocorrelation. This indicates the absence of second-order or higher-order autocorrelation in the model's residuals. Moreover, the results of the Hansen test reveal a p-value surpassing 0.1, signifying the affirmation of the original hypothesis regarding the validity of instrumental variables. The regression results remained reliable after accounting for endogeneity in the econometric model. The outcomes of the AR(1), AR(2), and Hansen tests indicate the instruments are valid and there is no significant instrument proliferation.

Overall, the SYS-GMM methodology, along with the other empirical models, provides rigor in estimating the effects of agricultural socialized services on grain production growth. The tests help control for endogeneity and strengthen the causal interpretation of the results. While limitations exist, the study applies best practices to ensure the models are sound. The collective empirical evidence from the various methods substantiates the conclusion that enhancing agricultural socialized services positively contributes to steady increases in grain production.

#### 4.2.2 Robustness test

To enhance the robustness of the findings, three distinct methodologies were employed. First, in recognition of the issuance of the “Strategic Plan for Rural Revitalization (2018–2022)" by the Central Committee and the State Council (CPC) in 2018, with a specific focus on addressing the “three rural issues,” which is anticipated to impact the stable and increased production of grain, the time interval was shortened, and the sample timeframe was narrowed from 2007 to 2017. Subsequently, a regression analysis was conducted anew. Second, acknowledging that a crucial manifestation of stable and increased grain production is the rise in actual grain production, a key factor in ensuring food security, a decision was made to use a single indicator—grain production input—rather than a comprehensive measure for stable and increased grain production. Consequently, the explained variable was replaced, and a regression analysis was re-executed. Thirdly, recognizing the potential time lag in the effect of agricultural socialized services due to the prolonged nature of food production, the article undertook a regression analysis employing the lagged variable of agricultural socialized services as the explanatory variable. The outcomes of these three robustness tests are detailed in columns (2)–(4) of Table 6. When compared to the results from the reference regression discussed earlier, the positive impact of enhancing agricultural socialized services on grain yield remains consistently evident. This reaffirms the fundamental conclusion of this study.

**Table 6:** Results of endogeneity and robustness tests.

Variables and parameters	SYS-GMM	Shortened year interval	Replace the explained variable	Explanatory variable lag by one phase
	(1)	(2)	(3)	(4)
L.PI	0.4957*** (0.1772)			
ASS	0.4022* (0.2145)	0.3538** (0.1664)	0.5546 ** (0.2195)	0.4394*** (0.1330)
Constant	0.3964** (0.3965)	0.2491* (0.1270)	0.2651*** (0.0774)	0.2578* (0.1496)
Control variable	Yes	Yes	Yes	Yes
Regional fixed effect	Yes	Yes	Yes	Yes
Time control effect	Yes	Yes	Yes	Yes
R^2^		0.4538	0.5974	0.5206
AR(1)	[0.016]			
AR(2)	[0.289]			
Hansen	[0.208]			
N	403	341	434	403

The values in the square brackets belong to the AR(1), and AR(2). The given values in the parentheses (.) are the standard errors. Hansen Test in the SYS-GMM regression corresponds to the *P* values of the 1st-order autocorrelation, 2nd-order autocorrelation, and Hansen over-identification test. ***, ** and * represent the level of significance of parameters at 1%, 5% and 10%, respectively.

### 4.3 Heterogeneity test

In diverse grain production environments, enhancements in agricultural socialized services can manifest unique positive externalities. Moreover, variations in grain industry structures, agricultural infrastructure, and the extent of “grain-oriented” land utilization across different regions may influence the efficacy of agricultural socialized services. Consequently, this study conducts group regression analyses utilizing data from both non-grain-producing areas and major grain-producing areas. The objective is to scrutinize the differentiated impact of agricultural socialized services on grain yield within varying grain production environments.

As illustrated in Table 7, discernible variations exist in the impact of agricultural socialized services on grain yield across different regions. In non-major grain-producing areas, these services exhibit a markedly positive and statistically significant effect on grain yield at the 1% significance level. Conversely, in major grain-producing areas, a positive impact is observed, although the significance level is lower at 10%. The rationale behind these distinctions can be attributed to several factors. Non-major grain-producing areas typically contend with less favorable production environments, employ small-scale production models, and witness lower enthusiasm among farmers for grain production. In such contexts, the augmentation of agricultural socialized services assumes a pivotal role in enhancing grain yield. These services adeptly integrate various grain production factors, optimize production methodologies, and consequently elevate grain yield. Moreover, they contribute to revitalizing farmers' enthusiasm for production and fostering the “grain-oriented” utilization of agricultural land, thereby exerting a significant marginal effect on grain yield in these regions.

**Table 7:** Regional heterogeneity test.

Variables and parameters	Nationwide	Major grain-producing areas	Non-major grain-producing areas
	(1)	(2)	(3)
ASS	0.3984*** (0.1086)	0.3913* (0.1861)	0.4668*** (0.1416)
Constant	0.2638** (0.1271)	0.4512** (0.1723)	0.2629 (0.1512)
Control variable	Control	Control	Control
Regional fixed effect	Yes	Yes	Yes
Time-fixed effect	Yes	Yes	Yes
R^2^	0.5371	0.3370	0.4500
N	434	182	252

***, **, and * represent the level of significance of parameters at 1%, 5%, and 10%, respectively. Standard errors are given in parentheses.

In major grain-producing regions, well-established agricultural infrastructure and industrial networks enable efficient grain production systems. While advances in agricultural socialized services can effectively supplement these developed grain ecosystems, the marginal boost to output is modest given the already high baseline productivity. Conversely, in areas with less organized agricultural support environments, strengthening specialized services can profoundly transform fragmented, smallholder farms into integrated modern grain operations. The marginal productivity gains are therefore much larger. The level of responsiveness in grain yield to agricultural service improvements depends on the existing conditions and institutional maturity of regional sectoral systems. Where foundational elements are robust, stepwise enhancements generate smaller, yet additive, value. Where significant restructuring is essential, the developmental impact from services infusion is far-reaching.

### 4.4 Influence mechanism test

To validate the mediating role of large-scale agricultural land management (H2), this study uses a formal stepwise regression approach based on the causal steps method [62]. This method allows for incrementally testing each relationship in the hypothesized mediation mechanism – from the independent variable (agricultural socialized services) to the mediator (large-scale land management) to the outcome variable (grain production growth). The stepwise regression approach quantifies the magnitude of the indirect effect and enables examining whether agricultural socialized services facilitate grain production increases through encouraging large-scale land management practices. While assuming linear relationships between variables, this mediation analysis provides empirical rigor in validating the proposed transmission channel. The test results align with the hypothesis that enhancing agricultural socialized services promotes grain production growth in part by driving large-scale agricultural land management.

Results found that agricultural socialized services have a positive influence on large-scale agricultural land management and grain yield. As shown in Table 8 column (2), the estimated coefficient for the impact of agricultural socialized services on large-scale land management is 1.5424, indicating these services significantly improve large-scale operations. Furthermore, column (3) reveals the internal relationships among the variables. The coefficients for agricultural socialized services and large-scale land management on grain yield are 0.3339 and 0.0042 respectively, both are statistically significant. The mediating effect share of large-scale land management is 16.26%. The empirical results validate the mediating pathway in which advancing agricultural socialized services spurs the implementation of large-scale farming techniques, subsequently elevating grain outputs. These findings lend credence to the postulated mechanism of indirect impact, whereby agricultural service improvements promote steady grain yield growth by enabling the consolidation of cultivated land parcels into aggregated, efficiency-enhancing production units.

**Table 8:** Results of mediating effect test.

Variables and parameters	Steady growth in grain yield	Large-scale management of agricultural land (SCA)	Steady growth in grain yield
	(1)	(2)	(3)
SCA			0.0418* (0.2185)
ASS	0.3984*** (0.1086)	1.5424** (0.0589)	0.3339*** (0.1181)
Constant	0.2638** (0.1271)	0.3070* (0.0249)	0.2510* (0.1308)
Control variable	Yes	Yes	Yes
Regional fixed effect	Yes	Yes	Yes
Time control effect	Yes	Yes	Yes
R^2^	0.5371	0.0318	0.6207
N	434	434	434

***, ** and * represent the level of significance of parameters at 1%, 5% and 10%, respectively. Standard errors are given in parentheses.

To evaluate the robustness of the mediation analysis, a bootstrap resampling technique with 1000 iterations is utilized to re-examine the direct and indirect pathways. As shown in Table 9, both effects remain significantly positive across the replications, as evidenced by p-values below 0.001, passing a strict 1% significance threshold. Moreover, the 95% confidence interval excludes zero. Collectively, these results demonstrate durable statistical validation of the intermediary role of large-scale farmland consolidation in enabling agricultural service advancements to translate into heightened grain outputs. These further cements the study hypothesis that agricultural socialization service development can indirectly but substantially raise grain production by facilitating scaled on-farm operations.

**Table 9:** Bootstrap method test for mediating effect.

Bootstrap Method	Estimated coefficient	Standard deviation	Z-value	P-value	95% confidence interval	
					Lower limit	Upper limit
Indirect effect	0.0814	0.0207	3.93	0.0000	0.0410	0.1220
Direct effect	0.4010	0.0295	13.90	0.0000	0.3520	0.4676

### 4.5 Threshold regression analysis

In this section, we investigated the non-linear effects of large-scale agricultural land management on grain yield increase within various levels of agricultural socialized services. Before delving into the threshold effect analysis, we first verified the number of threshold values and conducted significance tests on single, double, and triple thresholds using the threshold model. Subsequently, we employed the Bootstrap Method, conducting 500 rounds of repeated sampling. Table 10 provides insights into a threshold effect analysis, focusing on the variable ASS. The analysis is conducted under three distinct scenarios: Single threshold, Double threshold, and Triple threshold. In the case of a Single Threshold, the identified threshold number is 31.9900. The associated F-value is 0.0200, and the corresponding P-value is also 0.0200. The critical values at various significance levels are as follows: 1% (22.8774), 5% (27.7456), and 10% (34.5039). Moving on to the Double Threshold scenario, the threshold number is 20.7400. The F-value stands at 0.1140, and the P-value is 0.1140. Critical values for significance levels of 1%, 5%, and 10% are 21.6717, 26.4059, and 34.3350, respectively. Lastly, in the Triple Threshold scenario, the identified threshold number is 17.2600. The associated F-value is 0.6520, and the P-value is also 0.6520. Critical values at significance levels of 1%, 5%, and 10% are 42.1801, 48.5667, and 58.9731, respectively. These results signify the critical threshold values at which a significant change or effect is observed in the variable ASS. The F-value and P-value aid in assessing the statistical significance of the identified thresholds, while the critical values at different significance levels provide benchmarks for their evaluation.

**Table 10:** Threshold effect.

Threshold variable	Threshold number	F-value	P-value	Critical value		
				1%	5%	10%
ASS	Single threshold	31.9900	0.0200	22.8774	27.7456	34.5039
	Double threshold	20.7400	0.1140	21.6717	26.4059	34.3350
	Triple threshold	17.2600	0.6520	42.1801	48.5667	58.9731

**Fig. 1:**
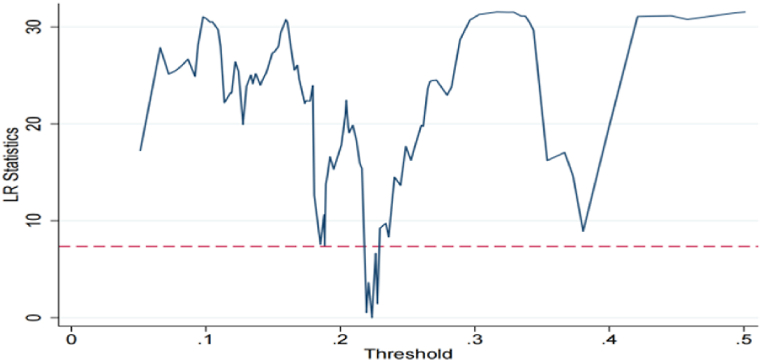
The function of the threshold likelihood ratio.

To further validate the accuracy of the threshold values, this study employs likelihood ratio (LR) statistics for verification. Fig. 1 illustrates the maximum likelihood ratio function corresponding to the threshold value, with the dashed line representing the critical value of the likelihood ratio statistic at the 5% significance level. As depicted in the figure, the threshold value falls within the associated confidence interval, and the likelihood ratio is below the critical value for a 5% significance level. Consequently, the threshold value aligns with the true value, ensuring its reliability.

Having confirmed the threshold effect, further analysis with 2007–2020 national data evaluates hypothesis H3 on the contingent relationship between large-scale farmland management and grain output across varying service settings. As shown in Table 11, agricultural service levels determine differential scaling effects on productivity. Below the 0.2236 threshold, the empirical association between land consolidation and yield ceases to hold, whereas, above the threshold, significantly positive returns emerge. This divergence stems from structural hindrances suppressing scale efficiencies under constrained service environments – notably around labor exchange, technology adoption, capital access, and dampened farmer enthusiasm from high coordination friction. However, in service-rich settings, these bottlenecks are mitigated through specialization, mechanization, accelerated resource flows, and improved cost-benefit viability of large-operational formats – catalyzing agricultural productivity. While expanded land size supports yield growth, critical thresholds in technical and institutional support must first be crossed to activate scale as an efficiency lever when traversing fragmented farm systems. The documented non-linearity spotlights agricultural services as the enabling backdrop for sustainable smallholder transformation through land aggregation.

**Table 11:** Regression results of threshold effect.

Variables and parameters	Steady growth in grain yield
SCA (ASS＜0.2236)	0.0162 (0.0533)
SCA (ASS≥0.2236)	0.0767** (0.0448)
Control variable	Control
Regional fixed effect	Control
Time-fixed effect	Control
R^2^	0.2909
N	434

Standard errors are given in parentheses. ** represents the level of significance of the parameter at 5%.

## 5 Conclusion and policy implications

### 5.1 Conclusion

The main aim of this study is to investigate the role of agricultural socialized services in enhancing farmland production in China. Utilizing panel data from 2007 to 2020, methods such as the Entropy Method, static panel models, mediator models, and threshold regression models were employed. The study found a significantly positive correlation between the enhancement of agricultural socialized services and increased grain production, attributed to various facets like agricultural means of production services, sci-tech information services, and social public services. Additionally, the mediating effect of large-scale agricultural land management and the presence of a single threshold effect related to the level of agricultural socialization services were identified.

### 5.2 Policy implications

Based on the results of the study, the following policy implications have been proposed.

1. Nations should prioritize investments in agricultural service ecosystems to enhance smallholder grain farming productivity, efficiency, and sustainability. This entails financing schemes, public-private incentives, extension programs, and targeted subsidies that provide small farms with accessible services spanning mechanization, market linkages, logistics, and certification. Robust support systems tailored to local contexts can unlock the potential of smallholders to advance food security.
2. Agricultural policy initiatives aimed at scaling up large-scale farming models should integrate coordinated support for upgrading specialized agricultural services. This can leverage synergies between consolidated operations and the availability of services tailored to user needs.
3. Innovation pilots testing new service delivery methods such as digital applications, farm machinery leasing models, and end-to-end logistics should inform broad policy frameworks for enabling such solutions through appropriate regulations, financial incentives, and public-private collaborations.
4. Development cooperation efforts around agricultural advancement between nations should encompass technical assistance and joint investments in context-specific agricultural service ecosystems, as part of food security aid commitments and programs along major growth corridors.
5. Multilateral agencies have a key role in facilitating global knowledge exchange on locally compatible agricultural service models that sustainably improve grain farming productivity and farmer livelihoods. Supported through such cooperative platforms, tailored solutions can be scaled for systemic impact.

The combined implications highlight the need for coordinated, multi-stakeholder efforts spanning micro pilots to macro policies across borders to unlock the potential of agricultural services in raising productivity, efficiency, and food security through grain farming systems worldwide. This study contributes to the understanding of the dynamic relationship between agricultural socialized services and farmland production in China. The findings underscore the importance of these services in not only increasing grain production but also in enhancing the overall efficiency and sustainability of agricultural practices. However, the study is not without limitations. The reliance on secondary data and the potential for regional variations in the impact of these services suggest the need for more localized studies. Future research could explore the differential effects of agricultural socialized services in various regions of China and other countries with similar agricultural contexts. It would also be beneficial to conduct longitudinal studies to observe the long-term impacts of these services on grain production and farmer welfare.

## Has data associated with your study been deposited into a publicly available repository?

No.

## Data availability

Data will be made available on request.

## Funding statement

The study is financially supported by the Taishan Young Scholar Program (tsqn202103070), the Taishan Scholar Foundation of Shandong Province, China.

## CRediT authorship contribution statement

**Aimin Wu:** Formal analysis, Data curation. **Ehsan Elahi:** Supervision, Resources, Investigation, Funding acquisition. **Fengtong Cao:** Writing – original draft, Validation. **Mohammad Yusuf:** Writing – review & editing, Validation, Supervision. **Mohammad Ilyas Abro:** Writing – review & editing, Formal analysis, Conceptualization.

## Declaration of competing interest

The authors declare that they have no known competing financial interests or personal relationships that could have appeared to influence the work reported in this paper.
